# Effects of transcranial direct current stimulation on cough reflex and urge-to-cough in healthy young adults

**DOI:** 10.1186/s12931-022-02020-x

**Published:** 2022-04-21

**Authors:** Peijun Gui, Lin Wang, Liya Guo, Chunwei Wu, Bo Zhang, Chen Chen, Ying Xie

**Affiliations:** Department of Rehabilitation Medicine, Beijing Friendship Hospital, Capital Medical University, 95 Yongan Road, Beijing, 100050 China

**Keywords:** Transcranial direct current stimulation, Dorsolateral prefrontal cortex, Cough reflex threshold, Urge-to-cough

## Abstract

**Background:**

Chronic cough is prevalent in the clinic. The existing therapies are mostly limited to medical treatment, with poor curative effects and serious side effects. Studies have suggested that the right dorsolateral prefrontal cortex (rDLPFC) may play an active role in the inhibitory pathway of cough elicitation. Thus, this study explored the effect of transcranial direct current stimulation (tDCS) on the rDLPFC activation in relation to cough reflex and urge-to-cough sensitivity.

**Methods:**

Twenty-three healthy young adults completed the experiment. Participants randomly received tDCS anodal stimulation, cathodal stimulation, and sham stimulation, and the interval between every two stimuli was at least one week. The tDCS (2 mA, 30 min) stimulated brain rDLPFC region. After tDCS intervention, cough reflex threshold and urge-to-cough were evaluated immediately by inhalation of citric acid-saline solution.

**Results:**

Compared with sham stimulation, the cough reflex thresholds logC_2_ and logC_5_ have increased under tDCS anodal stimulation (1.78 ± 0.55 g/L vs. 1.57 ± 0.57 g/L, p < 0.05; 1.92 ± 0.53 g/L vs. 1.67 ± 0.56 g/L, p < 0.05), accompanied by the increase of the urge-to-cough threshold LogC_u_ (0.76 ± 0.53 g/L vs. 0.47 ± 0.44 g/L, p < 0.05). In contrast, the urge-to-cough sensitivity expressed as UTC slope was not significantly changed (1.21 ± 0.86 point·L/g vs. 1.00 ± 0.37 point·L/g, p > 0.05), and there were no apparent changes in cough reflex thresholds Log C_2_ and logC_5_, urge-to-cough threshold LogC_u_, and urge-to-cough sensitivity UTC slope under tDCS cathodal stimulation, compared with sham stimulation.

**Conclusions:**

This study found that anodal tDCS stimulation of rDLPFC could significantly decrease cough reflex sensitivity, accompanied by the increase of urge-to-cough threshold. Further investigations targeting different brain regions using multiple central intervention techniques to explore the underlying mechanisms are warranted.

*Trial registration* The study protocol was registered for the clinical trial in China (registration number: ChiCTR2100045618)

## Background

Chronic cough, lasting for more than 8 weeks, is a frequently occurring symptom in about 10% of the general adult population worldwide [1]. Persistent long-term coughing can considerably impact patients' physical and psychological health and overall quality of life as well [2]. There have been a large number of outpatients visiting the clinic every year due to seasonal coughing, which sometimes remains difficult to be completely cured. Although cold-medicine based therapy is the primary treatment option, however, the curative effect is limited and often accompanied by adverse side-effects [3]. Therefore, non-pharmaceutical treatment options are gradually coming into the limelight as an alternative approach.

The cough reflex is usually thought to be mediated at the brainstem level. Studies have demonstrated that people often sense a stimuli-induced motivation of cough-like symptoms to protect their respiratory airway, which is termed the urge-to-cough. Importantly, urge-to-cough is a critical component of the sustained motivation that mediates the cognitive responses of airway stimulation prior to coughing [4]. The intensity of the urge-to-cough has been positively correlated with that of actual cough. Furthermore, the decrease in the intensity of urge-to-cough is often accompanied by the increased cough reflex threshold [5]. Mazzone et al. [6] have found the cerebral cortex and subcortical areas, such as the insula cortex, anterior midcingulate cortex, primary sensory cortex, orbitofrontal cortex, supplementary motor area, and cerebellum, are involved in the regulations of the urge-to-cough as well as cough reflex. Comparison of the central neural responses to airway stimulation between healthy and cough hypersensitive subjects using functional Magnetic Resonance Imaging (fMRI) has revealed that patients with cough hypersensitivity are characterized by central amplification of cough sensory inputs and reduced capacity to suppress cough-associated motor behaviors [7]. Cough reflex and urge-to-cough sensitivity may be directly influenced by the regulatory cortical activity.

Transcranial direct current stimulation (tDCS) is a noninvasive brain stimulation technique to regulate the activity of cerebral cortical neurons. It can modulate neural plasticity and regulate cerebral cortex function by applying mild direct current to the scalp through one or two electrodes placed thereon. Anodal stimulation can induce neuronal depolarization, thus improving the excitability of the cortical neurons, while cathodal stimulation exerts exactly the reverse effects [8]. tDCS has been broadly used in various clinical fields. For example, Bornheim et al. [9] applied tDCS to stroke patients by inducing anodal stimulation at stroke lesions in the primary motor cortex. Therapeutically, the patient receives tDCS intervention (2 mA, 20 min) 5 times per week for 4 weeks, combined with traditional rehabilitation treatment. It has been found that the motor and somatosensory functions of stroke patients significantly improve after tDCS therapy. Moreover, Suntrup-Krueger et al. [10] applied tDCS, with a current intensity of 1 mA for 20 min each time, to the contralesional motor cortical areas in stroke patients with acute dysphagia. The results showed significant improvement in swallowing function after tDCS intervention for four consecutive days.

Whether the cough reflex and urge-to-cough sensitivity of subjects can be modulated by tDCS intervention remains unclear to date. Studies have shown that tDCS can significantly improve swallowing dysfunction and pain [11, 12], since the cough reflex, swallowing reflex, and pain mechanism operate under the same central regulatory hub in the brain [13, 14]. Thus, we speculated that tDCS might influence individual’s cough reflex. Furthermore, studies have shown that right dorsolateral prefrontal cortex (rDLPFC) brain activity is simultaneously induced when the individual’s urge-to-cough decreases, suggesting that rDLPFC may play a vital role in the inhibitory pathway of cough symptoms [15]. Similarly, the placebo analgesia studies reported elevated brain activity in the dorsolateral prefrontal cortex, suggesting an essential role the region may play in regulating sensorimotor responses in the brain [16]. To date, there is no study exploring the effects of tDCS on rDLPFC stimulation related to cough reflex sensitivity. In this study, two stimulation protocols, anodal and cathodal stimulation, were followed to conduct the investigation on the rDLPFC with sham stimulation used as a control to reveal the effects of the two different tDCS modes on the cough reflex and urge-to-cough sensitivities.

## Methods

### Subjects

Initially, 25 healthy young adults, including 14 males and 11 females, were enrolled in this study. After one male and one female participant dropped out, 23 adults were finally recruited to this trial. All participants were initially recruited via WeChat on the campus of Capital Medical University. The mean age was 22.5 ± 3.6 (S.D.) years. Subjects without a history of respiratory diseases, respiratory infection, and/or seasonal allergy within past 4 weeks were selected. Participants having menstruation, pregnancy, or lactation, had metal implantation, had a history of neuropsychiatric diseases, alcohol or drug abuse were excluded from this study. All participants signed written informed consent for their participation in this study. The study protocol was approved by the review board of the medical ethics committee of Beijing Friendship Hospital affiliated with Capital Medical University (approval number: 2021-P2-014-02) and registered for the clinical trial in China (registration number: ChiCTR2100045618).

### Cough reflex threshold and urge-to-cough

Citric acid was selected as a tussive agent for the cough reflex test. Firstly, citric acid was dissolved in 0.9% saline solution. The initial concentration was 0.7 g/L, providing a two-fold incremental concentration to 360 g/L. An ultrasonic nebulizer (NE-C900, Omron medical devices Co Ltd, Beijing, China) delivered a tidal nebulized citric acid solution. The mean mass median diameter of the particles generated by the nebulizer was 3.0 ± 1.0 µm, and the output speed was 0.25 mL/min. Each subject inhaled physiologic saline as the control and then inhaled the citric acid solution in a progressively increasing concentration until five or more coughs were elicited. Each inhalation time was set to 1 min, and the interval between two consecutive inhalations was 2 min. The number of coughs was counted by technicians blinded to the experimental grouping and the study purpose. The lowest citric acid concentrations that elicited two or more coughs and five or more coughs were defined as the lower and upper cough reflex thresholds, recorded as C_2_ and C_5,_ respectively. The maximum citric acid concentration that caused zero time of cough was recorded as C_0max_.

Each subject was assessed for the urge-to-cough sensitivity using a modified Borg scale immediately after completion of each inhalation. The Borg scale ranges from no need-to-cough (0) to maximum urge-to-cough (10). The urge-to-cough scale was placed in front of the subjects, and subjects were instructed to point to the corresponding scale score based on their actual feelings, which the experimenter recorded. In order to evaluate the intensity of urge-to-cough, subjects were required to ignore other feelings, such as dyspnea, burning in the throat, choking. During the inhalation of citric acid, subjects were made aware of the fact that their sensitivity to urge-to-cough could increase, decrease, or remain unchanged, and their Borg score should reflect the degree of sensitivity. According to previous studies [5], there is a linear correlation between the Borg score of urge-to-cough and the concentration of citric acid using log–log transformation. Therefore, we used linear regression analysis to calculate the slope on a log–log scale. The citric acid concentration corresponding to the first Borg scale score of not 0 was defined as the urge-to-cough threshold, recorded as C_u_.

### Transcranial direct current stimulation (tDCS)

**Fig. 1:**
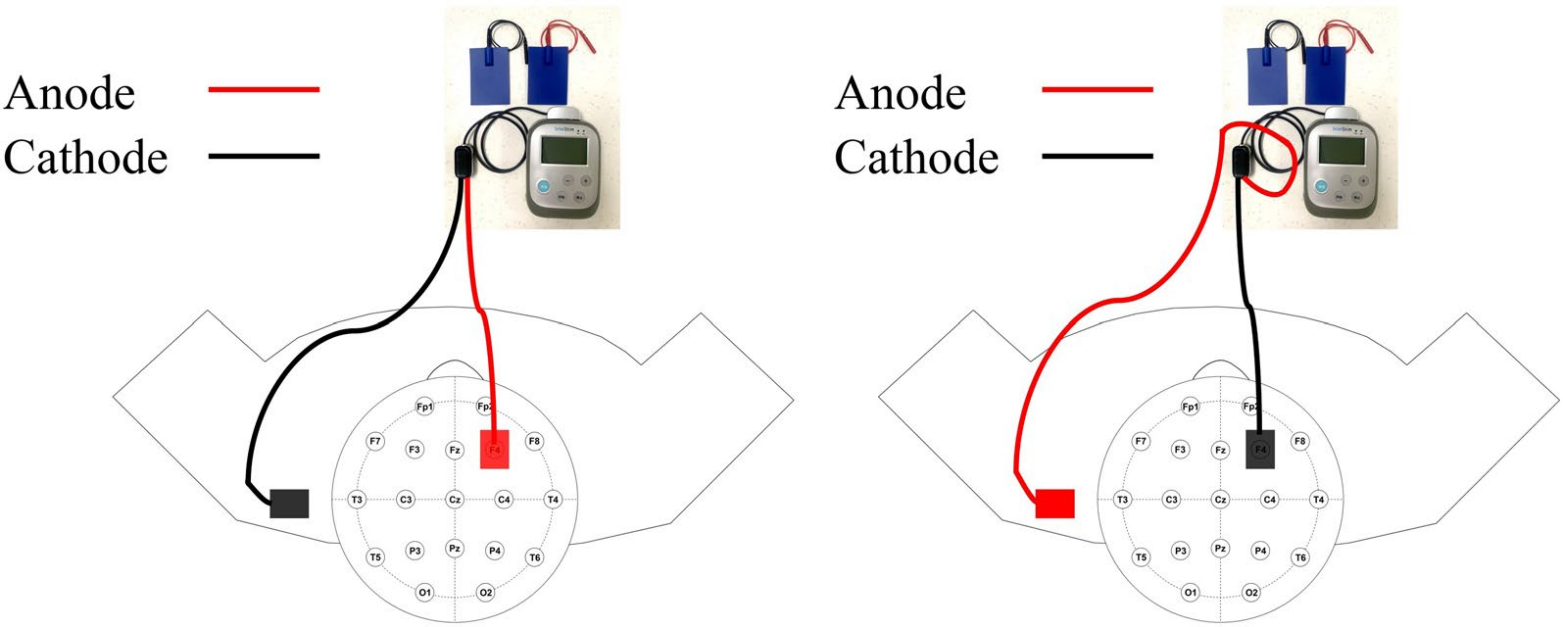
Transcranial direct current stimulation electrode position

Direct current was applied using a saline-soaked pair of surface sponge electrodes (5 × 7 cm^2^) and delivered using a battery-driven, constant current stimulator (IS200, Zhineng Co. Ltd, Chengdu, China) [17]. A constant current of 2 mA (0.057 mA/cm^2^) was applied for 30 min. There were three different stimulation modes. In mode 1 (the anodal mode), the anode was placed on the rDLPFC, and the cathode on the left shoulder (Fig. 1). In mode 2 (the cathodal mode), the cathode was placed on the rDLPFC, and the anode on the left shoulder (Fig. 1). In mode 3 (the sham mode), anode and cathode were randomly placed on the rDLPFC and the left shoulder. The stimulator produced electricity only for the first 30 s. The current intensity was gradually increased in the initial 15 s and then gradually decreased in the following 15 s. There was no current in the rest time. According to the 10–20 system 32-lead electrode distribution position, the rDLPFC corresponded to F4, so that the top center was 8 cm forward along the sagittal line, and then a vertical line on the sagittal section was drawn at this point, with a side opening of 6 cm.

### Experimental protocol

The researcher introduced the whole experimental procedure to all subjects before it started. All subjects first underwent a pulmonary function examination and then randomly received tDCS interventions in three different stimulation modes, namely anodal stimulation (anodal mode), cathodal stimulation (cathodal mode), or sham stimulation (sham mode). Immediately after the tDCS intervention, the subjects underwent citric acid challenge to evaluate their cough reflex thresholds and urge-to-cough sensitivities. In order to avoid the sustained effect of tDCS, the interval between two consecutive stimulation modes was at least one week. Previous studies have reported that [18] people may experience adverse effects after tDCS intervention, such as dizziness, headache, neck pain, and scalp burning. Therefore, subjects were asked whether they had any uncomfortable sensations after each intervention.

### Data analysis

Data are expressed as mean ± SD except otherwise stated. The group variances were tested using the Shapiro–Wilk normality test and found no significant difference, other than urge-to-cough log–log slope. The paired sample t-test and Wilcoxon test were used to compare anodal stimulation or cathodal stimulation with sham stimulation. A p < 0.05 was considered significant.

To determine the sample size for the study, a pilot trial of six subjects were enrolled to evaluate both cough reflex sensitivity and UTC. Based on the pilot trial results, the sample size was calculated by the difference in LogC_5_ between the anodal and sham groups by PASS 11.0. With a power of 0.9 and a significance level of 0.05, the sample size was calculated to be sufficient with 18 participants. With uncertainty about variability and an expected drop-out rate of 10%, we aimed to include 20 healthy young adults. We finally recruited 23 healthy young adults.

## Results

All 23 subjects completed the experiment without any difficulty or side effects. The characteristics of subjects are summarized in Table 1. All subjects had normal pulmonary functions.

**Table 1:** Baseline characteristics of subjects

	N = 23
Age (years)	22.48 ± 3.60
Male/Female (n)	13/10
Height (cm)	171.52 ± 8.46
Weight (Kg)	65.63 ± 10.95
FEV1 (L)	3.91 ± 0.68
FEV1 (% predict)	101.60 ± 9.42
FVC (L)	4.62 ± 0.99
FVC (% predict)	108.60 ± 13.02
FEV1/FVC (%)	85.47 ± 6.26

Data are represented as mean ± S.D

**Fig. 2:**
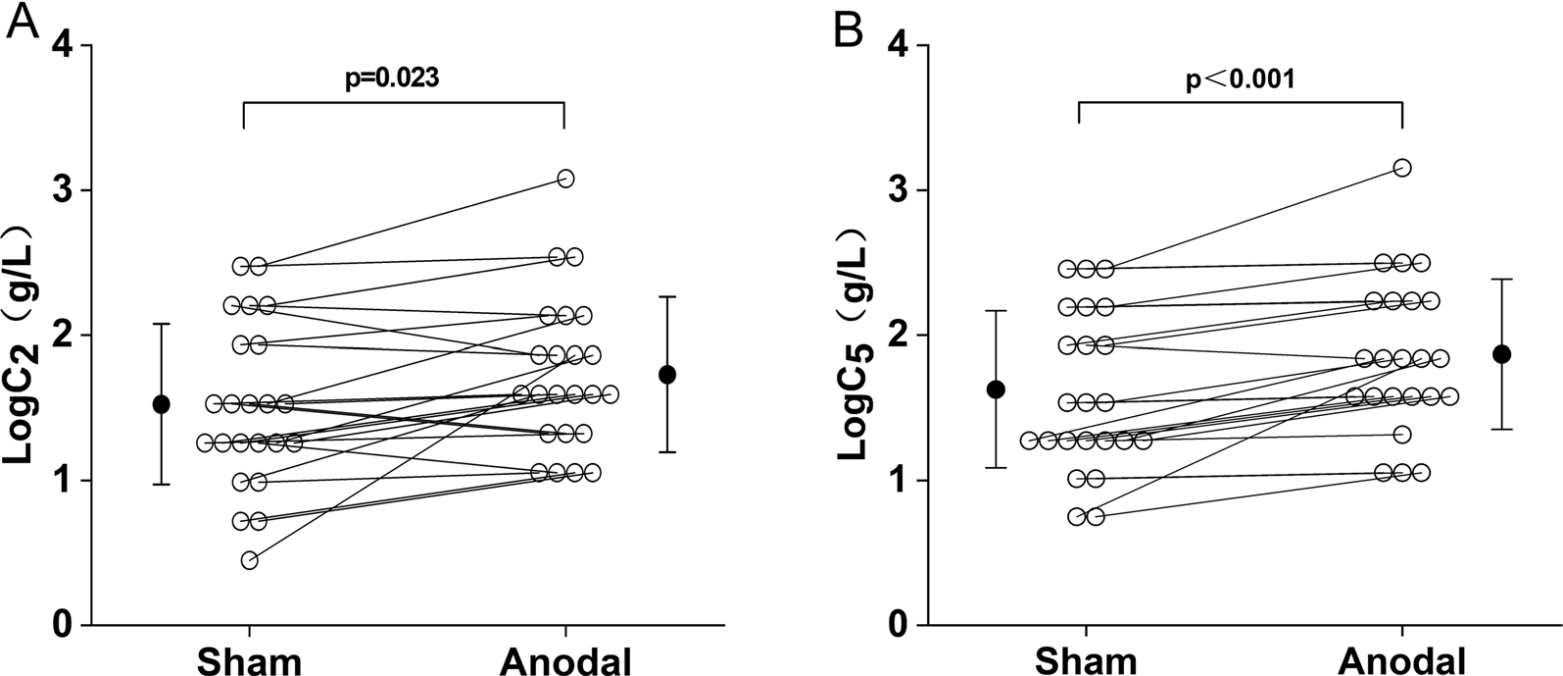
Comparison of cough reflex threshold between the sham and anodal modes. **A** LogC_2_ in each group. **B** LogC_5_ in each group. Open circles indicate the value of each subject. Closed circles and error bars indicate the mean and S.D. in each group, respectively. LogC_2_ = the log transformation of the lowest concentration of citric acid that elicited two or more coughs. LogC_5_ = the log transformation of the lowest concentration of citric acid that elicited five or more coughs

**Fig. 3:**
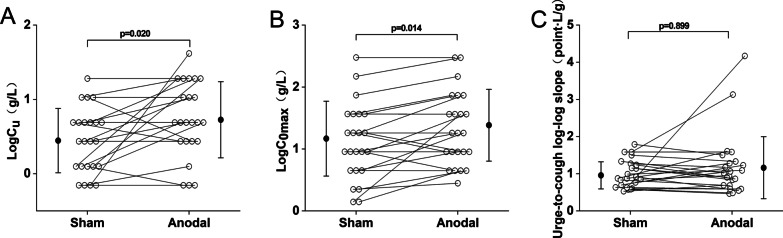
Comparison of urge-to-cough (UTC) between the sham and anodal modes. **A** LogC_u_ in each group. **B** LogC_0max_ in each group. **C** The UTC log–log slope in each group. Open circles indicate the value of each subject. Closed circles and error bars indicate the mean and S.D. in each group, respectively. LogC_u_ = the log transformation of concentration of citric acid at a threshold of UTC. LogC_0max_ = the maximum concentration of citric acid causing zero time of cough. UTC log–log slope = the slope between UTC scores and citric acid concentration on a log–log scale

Figure 2 shows the comparison of cough reflex threshold under the anodal mode and the sham mode, as expressed by LogC_2_ and LogC_5_. The LogC_2_ in the anodal mode shown in Fig. 2A was significantly higher than that in the sham mode (1.78 ± 0.55 g/L vs. 1.57 ± 0.57 g/L, p < 0.05). The LogC_5_ between the anodal and sham modes was shown in Fig. 2B. Compared with the sham mode, the cough reflex threshold LogC_5_ in the anodal mode was also significantly higher (1.92 ± 0.53 g/L vs. 1.67 ± 0.56 g/L, p < 0.05). Figure 3 shows the urge-to-cough sensitivity between the anodal and sham modes. As shown in Fig. 3A, LogC_u_ in the anodal mode was significantly increased than that of the sham mode (0.76 ± 0.53 g/L vs. 0.47 ± 0.44 g/L, p < 0.05). Figure 3B shows LogC_0max_ of 1.19 ± 0.62 g/L, 1.42 ± 0.60 g/L for the sham and anodal modes, respectively. LogC_0max_ was significantly higher in the anodal mode than that for the sham mode (p < 0.05). As shown in Fig. 3C, the urge-to-cough log–log slopes were 1.00 ± 0.37 point·L/g, 1.21 ± 0.86 point·L/g in the sham mode and anodal mode, respectively. There was no significant difference between the two groups.

**Fig. 4:**
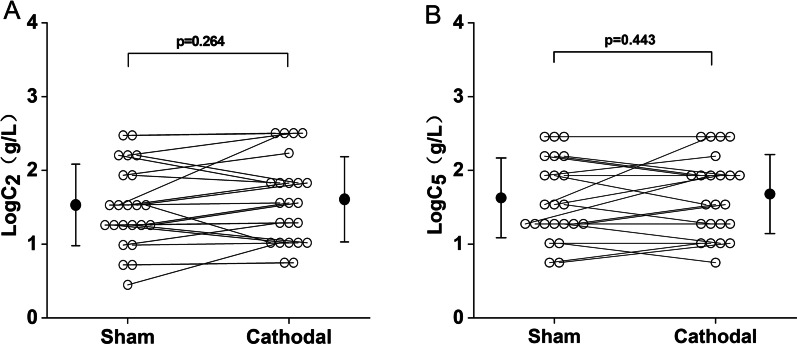
Comparison of cough reflex threshold between the sham and cathodal modes. **A** LogC_2_ in each group. **B** LogC_5_ in each group. Open circles indicate the value of each subject. Closed circles and error bars indicate the mean and S.D. in each group, respectively. LogC_2_ = the log transformation of the lowest concentration of citric acid that elicited two or more coughs. LogC_5_ = the log transformation of the lowest concentration of citric acid that elicited five or more coughs

**Fig. 5:**
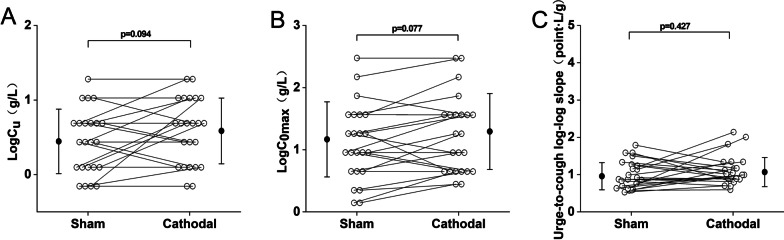
Comparison of urge-to-cough (UTC) between the sham and cathodal modes. **A** LogC_u_ in each group. **B** LogC_0max_ in each group. **C** The UTC log–log slope in each group. Open circles indicate the value of each subject. Closed circles and error bars indicate the mean and S.D. in each group, respectively. LogC_u_ = the log transformation of concentration of citric acid at a threshold of UTC. LogC_0max_ = the maximum concentration of citric acid causing zero time of cough. UTC log–log slope = the slope between UTC scores and citric acid concentration on a log–log scale

Figure 4 shows the comparison of cough reflex threshold under the cathodal mode and the sham mode, as expressed by LogC_2_ and LogC_5_. There was no significant difference in LogC_2_ between the cathodal and the sham modes (1.65 ± 0.60 g/L vs. 1.57 ± 0.57 g/L, p > 0.05), as shown in Fig. 4A. Similarly, no significant difference was observed in LogC_5_ between the cathodal mode and the sham mode (1.72 ± 0.55 g/L vs. 1.67 ± 0.56 g/L, p > 0.05), as shown in Fig. 4B. Figure 5 shows the urge-to-cough sensitivity between the cathodal mode and the sham mode. As shown in Fig. 5A, the difference in LogC_u_ was not significant between the cathodal and sham modes (0.62 ± 0.45 g/L vs. 0.47 ± 0.44 g/L, p > 0.05). Figure 5B shows LogC_0max_ of 1.19 ± 0.62 g/L and 1.33 ± 0.63 g/L for the sham and cathodal modes, respectively, and there was no significant difference in LogC_0max_ between the cathodal and sham modes. In Fig. 5C, the urge-to-cough log–log slopes were 1.00 ± 0.37 point·L/g and 1.11 ± 0.40 point·L/g in the sham and cathodal modes, respectively, and no significant differences was seen between the two groups.

## Discussion

Our study explored the effect of tDCS on the cough reflex. The results showed that single anodal tDCS applied to the rDLPFC could significantly increase the cough reflex threshold, accompanied by the increase in urge-to-cough threshold but not the sensitivity.

This study, for the first time, reported the effects of the direct intervention of the central cerebral cortex on cough reflex sensitivity and urge-to-cough. The cough reflex is mediated by the higher-order neuronal circuitry [19], including the activation and inhibition circuits [20]. The primary sensory cortex, orbitofrontal cortex, insula cortex, anterior midcingulate cortex, and other brain regions are involved in the activation circuit [6]. The rDLPFC and parietal cortex are likely to play a role in the inhibition circuit [15]. The activity of the rDLPFC decreases significantly in patients with cough hypersensitivity [7]. Studies on the mechanism of placebo antitussive therapy have found that the magnitude of the rDLPFC activation was significantly related to the magnitude of the placebo antitussive effect. Since the brain regions involved in cough and breathing suppression, including the right ventral inferior frontal gyrus, anterior insula, and the middle cingulate gyrus, exhibit no significant difference between the placebo and control groups [15]. Thus, it is suggested that the rDLPFC might play a vital role in the inhibition of cough. Moreover, pain and cough reflex activities share overlapping central mechanisms [21]. In addition, a study exploring the central mechanism of analgesia has found that the analgesic effect caused by the placebo is related to the increased activation of the bilateral DLPFC during harmful stimulation [22]. Given that noninvasive brain stimulation technology can produce an analgesic effect by stimulating the DLPFC [23]. Therefore, we speculated that the rDLPFC intervention through noninvasive brain stimulation technology might affect cough reflex and urge-to-cough sensitivity.

tDCS is a noninvasive brain stimulation technology, which applies mild direct current to the scalp to modulate cortical excitability. Previous studies have reported that tDCS (2 mA, 20 min, electrode size: 3 × 5 cm) can produce an analgesic effect by stimulating the left DLPFC [24]. A randomized controlled study also showed that after tDCS intervention in the swallowing motor cortex, the swallowing function of stroke patients was significantly improved [10]. Notably, pain, swallowing reflex, and cough reflex mechanisms overlap in the central regulatory pathway. When painful stimuli were applied to the volunteers, fMRI showed the activation of several brain regions, such as the prefrontal cortex, insular cortex, primary somatosensory cortex, similar to the brain regions activated when individual coughs [25]. Thus, tDCS stimulation of the cerebral cortex might also influence the cough reflex sensitivity. Our results showed that individuals’ cough reflex and urge-to-cough threshold were significantly increased after the anodal tDCS stimulation of the rDLPFC activity, indicating the possibility of modulating cough reflex through the regulation of the central nervous system (CNS).

In addition, our results further supported the active role of the rDLPFC upon anodal tDCS stimulation in the cough inhibition pathway. On the other hand, cathodal tDCS reduced rDLPFC neural activity but did not affect individuals’ cough reflex and urge-to-cough sensitivity. A systematic meta-analysis [26] has revealed that following the cathodal tDCS intervention, the behavioral results related to cognitive function may not change significantly. Several studies on the motor and a few studies on cognition have found that the inhibition can rarely be caused by the cathode tDCS [27–29]. One possible interpretation for the findings is that the bilateral interactions support the contralateral compensation [26]. When cathodal tDCS inhibits the neural activity in the stimulated cerebral hemisphere, corresponding brain regions in the contralateral cerebral hemisphere may be activated to maintain the stability of physiological functions [27]. Furthermore, brain regions in the cough activation pathway should be targeted for patients who need to improve urge-to-cough sensitivity, such as elderly patients with aspiration pneumonia, and the rDLPFC may be a potential target of central antitussive therapy in the future.

At present, antitussive therapy includes both pharmacological and nonpharmacological interventions. The traditional antitussive drugs mainly include codeine, morphine, gabapentin, pregabalin, amitriptyline, but the efficacy is poor and exhibits adverse effects. Although several studies have focused on identifying new targeted antitussive drugs for the peripheral pathway, however, these new antitussive drugs are mostly in the pre-clinical stage, and their clinical efficacies need to be further confirmed [3]. The existing nonpharmacological therapies mainly include health education, cough suppression, breathing training, vocal hygiene and hydration, psychological counseling, and laryngeal massage. Unfortunately, patients need long-term persistence to have effective therapeutic benefits, and only about half of those patients finally adhered to the treatment [30]. Hence, this study demonstrated a short-term therapeutic intervention strategy by applying anodal tDCS to the rDLPFC to inhibit cough reflex. Moreover, tDCS therapy is cheaper, portable, well-tolerated, and safer [31, 32]. Therefore, this technique may be clinically used as a new therapy to treat chronic cough in the future.

Despite these therapeutic advantages, there are also certain limitations in the study. (1) The subjects were all healthy young adults. Belvisi et al. showed that the reduction in experimentally evoked cough might not reliable to predict the antitussive effects [33]. Hence, whether tDCS can be applied to patients with chronic cough and cough hypersensitivity need further study. (2) Only one cough reflex inhibitory targeted brain region was investigated. Whether other brain regions are involved in cough reflex should be investigated further. (3) The long-term effects of treatment should be investigated.

## Conclusions

This study confirmed that anodal tDCS stimulation of rDLPFC could increase cough reflex threshold and urge-to-cough threshold significantly, but the urge-to-cough sensitivity exhibited no significant change. Further research is necessary to reveal the underlying mechanisms and develop novel intervention targets for the central nervous system.

## Data Availability

The datasets analyzed during the current study are available from the corresponding author on reasonable request.

## Abbreviations

**LogC**_**2**_: The log transformation of the lowest concentration of citric acid that elicited two or more coughs
LogC_5_: The log transformation of the lowest concentration of citric acid that produced five or more coughs
LogC_u_: The log transformation of concentration of citric acid at a threshold of urge-to-cough
LogC_0max_: The maximum concentration of citric acid causing zero time of cough
UTC Slope: Urge-to-cough log–log slope, the slope between urge-to-cough scores and citric acid concentration on a log–log scale
fMRI: Functional magnetic resonance imaging
tDCS: Transcranial direct current stimulation
rDLPFC: Right dorsolateral prefrontal cortex
